# Temporal Distinction between Male and Female Floral Organ Development in *Nicotiana tabacum* cv. Xanthi (Solanaceae)

**DOI:** 10.3390/plants9010127

**Published:** 2020-01-19

**Authors:** Hongli Chang, Fengjie Sun

**Affiliations:** 1 Shaanxi Key Laboratory for Animal Conservation, School of Life Sciences, Northwest University, Xi’an 710069, China; hlchang@nwu.edu.cn; 2 School of Science and Technology, Georgia Gwinnett College, Lawrenceville, GA 30043, USA

**Keywords:** *Nicotiana tabacum*, Solanaceae, tobacco, plastochron index, pistil, carpel, anther, stamen

## Abstract

Early floral developmental investigations provide crucial evidence for phylogenetic and molecular studies of plants. The developmental and evolutionary mechanisms underlying the variations in floral organs are critical for a thorough understanding of the diversification of flowers. Ontogenetic comparisons between anthers and pistil within single flowers were characterized over time in *Nicotiana tabacum* cv. Xanthi. The ages of 42 tobacco flower or flower primordia were estimated using corolla growth analysis. Results showed that the protodermal layer in carpel primordia contributes to carpel development by both anticlinal and periclinal divisions. Periclinal divisions in the hypodermal layer of the placenta were observed around 4.8 ± 1.3 days after the formation of early carpel primordia (ECP) and ovule initiation occurred 10.0 ± 0.5 days after ECP. Meiosis in anthers and ovules began about 8.9 ± 1.1 days and 14.4 ± 1.3 days after ECP, respectively. Results showed an evident temporal distinction between megasporogenesis and microsporogenesis. Flower ages spanned a 17-day interval, starting with flower primordia containing the ECP and anther primordia to the tetrad stage of meiosis in megasporocytes and the bicellular stage in pollen grains. These results establish a solid foundation for future studies in order to identify the developmental and molecular mechanisms responsible for the mating system in tobacco.

## 1. Introduction

In angiosperms, various factors affect the successful pollination and subsequent fertilization of the egg in the female gametophyte (i.e., embryo sac). The mating patterns are greatly influenced by the phenotypic variations in floral traits, such as herkogamy (the spatial separation of anthers and stigma) and dichogamy (the temporal separation of male and female phases) [1,2,3,4,5,6,7,8], cleistogamy (the self-fertilization within a permanently closed flower) [3,9], and heterostyly (styles of different lengths relative to the stamens in the flowers of different plants) [10,11]. The diversity of functional floral traits is defined by the genetic and developmental mechanisms controlling the differentiation of these reproductive organs during floral ontogeny. For example, studies have shown that the selfing flowers of *Arenaria uniflora* have evolved from the outcrossing ones by a reduced developmental rate and longer growth duration [4].

Selfing is commonly found in diverse groups of angiosperms and functions in different forms, including geitonogamy (pollen is transferred to a different flower on the same plant) [12,13], autogamy (pollen is deposited on the stigma of the same flower during floral development), and cleistogamy (obligated self-pollination in closed, bud-like flowers) [3,9]. The evolutionary transition from outcrossing to selfing has been widely studied in many systems [14]. Although selfing may have an advantage over outcrossing under various environmental and reproductive conditions [15,16], multiple mechanisms have been demonstrated to prevent inbreeding depression caused by selfing [17,18]. Studies have shown that the evolutionary changes in the gynoecium and androecium contribute directly to the determination of mating patterns. For example, the evolution of selfing depends on the variations in floral development underlying the developmental mechanisms of divergence in floral morphology [4,8], which is derived from heterochrony (the change in timing of floral development) [3,5,19], resulting either in phenotypically immature or smaller flowers, or mature flowers at smaller size resembling immature buds. Therefore, comparisons between female and male floral organ development over time will provide an essential foundation for understanding how integrated floral traits are modified through evolution [4,7,8,20,21]. Furthermore, heritable phenotypic variations in ontogeny function to determine the short-term potential for natural selection to cause evolutionary changes [22], making further evolution of the mating system possible. For example, the autogamous selfing may be derived from the reduction of the floral traits in both herkogamy and dichogamy, both of which are determined by the development of the floral organs. Studies have generally focused on the phenotypic variations in mature floral form, while investigations of ontogenetic variation underlying changes in floral traits, particularly both the gynoecium and androecium, are sparse [6,23]. *Nicotiana tabacum* is a self-compatible species with flowers arranged in an inflorescence type of cyme. To investigate the mechanism of nonrandom mating in this self-compatible species, Alves et al. [24] demonstrated that diverse intraspecific pollen-pistil interactions regulate the rates of pollen tube growth depending on the stigma and the mature transmitting tissues and eventually determine the fertilization and seed set. The genetic basis of self-compatibility, as well as self-incompatibility mating systems in *Nicotiana* [25] and *Prunus* [26], was investigated recently, demonstrating the molecular mechanisms of the recognition between the pollen and the stigma and the pollen tube growth.

From a molecular perspective, the timing and duration of gene expression during flower organogenesis is a fundamental component of genetic mechanisms controlling reproductive development [27,28,29]. Knowing the temporal expression pattern of the organ-specific genes in both the stamen and pistil helps elucidate the genetic mechanisms controlling the formation of a fully functional flower [30]. However, flower organogenesis is a continuous process, while the time-based studies reveal developmental rates of floral organs in ways that could be more informative than those of the size-based (e.g., flower bud length) allometric studies. For example, flower bud length and floral organ length were used to calculate the duration of different developmental stages, and a close correlation was demonstrated between flower bud length and the developmental stage of either anthers [31] or microspores [32]. However, the flower bud lengths and flower organ lengths cannot be measured nondestructively in young flowers or flower primordia, which must be aged using an alternative methodology, e.g., plastochron approach [33]. The genetic mechanisms coordinating floral organ initiation and development were explored in tomato [34]. Furthermore, MADS-box genes have been identified as the key components of gene regulatory networks involved in plant responses to plant developmental plasticity under various environmental conditions [35].

Plastochron, characterized as the time between the sequential initiations of the floral organs, is commonly applied as a measure of the duration of flora organ growth. Erickson and Michelini [33] developed the plastochron index (PI) to represent the physiological ages of plants and to quantify the morphological characteristics of plants, even at their early stages when growth cannot be observed. Based on the assumption that plant organs (1) have an exponential phase of growth, (2) grow at equal rates at successive nodes, and (3) initiate equally spaced in time (i.e., constant plastochron) [33], the PI provides a morphological time scale which is reliable for staging development and providing chronological ages of plant organs. Since its establishment, PI has been applied in various species in developmental studies to determine the ages of various plant, mainly floral, organs [36]. This method is further expanded to estimate the PI by an alternative approach when the rate of organ initiation does not remain constant [3,6]. Plastochron is determined to be an accurate and efficient method to detect the temporal patterns of ontogenesis among floral organs and consequently to make it possible to compare early stamen and pistil development over time.

In the present study, we estimate the ages of flowers or flower primordia in *Nicotiana tabacum* L. cv. Xanthi. In a typical flower of tobacco, the gynoecium is syncarpous containing one pistil with two fused carpels surrounded by five stamens. Our goals are to characterize the temporal developmental patterns in both female and male floral organs in real-time to determine the changes in floral ontogeny and the impact of these alternations on the mating system.

## 2. Results

### 2.1. Corolla Growth Analysis

Mature flowers of *Nicotiana tabacum* cv. Xanthi are white-pinkish with trumpet-shaped floral tubes, which are generally 5–6 cm in length and 5 mm in diameter, with expansion around the areas of both calyx and throat. The calyx has five narrowly triangular lobes.

The average cyme plastochrons of the six plants in Group 1 increased gradually from 1.45 days at flower position 2 to 2.85 days at position 8 with a minor decrease at flower position 6 (Table 1). The PIs of the six cymes in Group 2 plants increased gradually from 1.61 to 3.00 as the corolla length of the first flower in each cyme increased (Table 2).

### 2.2. Pistil and Stamen Development

**Figure 1:**
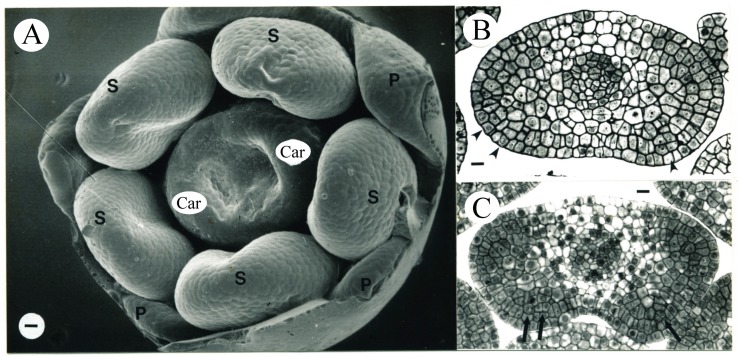
Scanning electron microscope (SEM) micrograph of a tobacco flower (**A**) and the transverse sections of anthers (**B**,**C**) following carpel initiation. This and subsequent SEM micrographs are made of epoxy casts. (**A**) Two carpel primordia (Car) initiated. This flower is corresponding to Stage –7, as described in [30]. This stage is here defined as the early carpel primordial (ECP) and is set at zero on the time scale for all of the other flower ages reported in the present study. S, stamen; P, petal primordium. Bar = 18 µm. (**B**) An anther from the flower shown in (**A**). Three recent periclinal divisions of L2 cells are evident (arrowheads). Bar = 14 µm. (**C**) Transverse section of a representative anther from the age interval 1.3 ± 1.2 days after ECP. Recent periclinal divisions of L2 cells are evident (arrows). Bar = 16.7 µm.

In the youngest floral primordium aged in this study, two carpel primordia elevated on the floral apex (Figure 1A), corresponding to Stage –7 by [30]. These two carpel primordia were defined as the early carpel primordia (ECP) and set equal to zero on the time scale. No differentiation of vascular tissue was observed in these two carpel primordia. The pistil was 0.49 mm in length. The stamen and anther developmental characteristics in our study and those reported previously [6,30] are listed in Table 3. Two flowers were observed on the second day (1.3 ± 1.5 days) after ECP with pistil lengths of 0.51 and 0.59 mm, respectively (Figure 1C).

**Figure 2:**
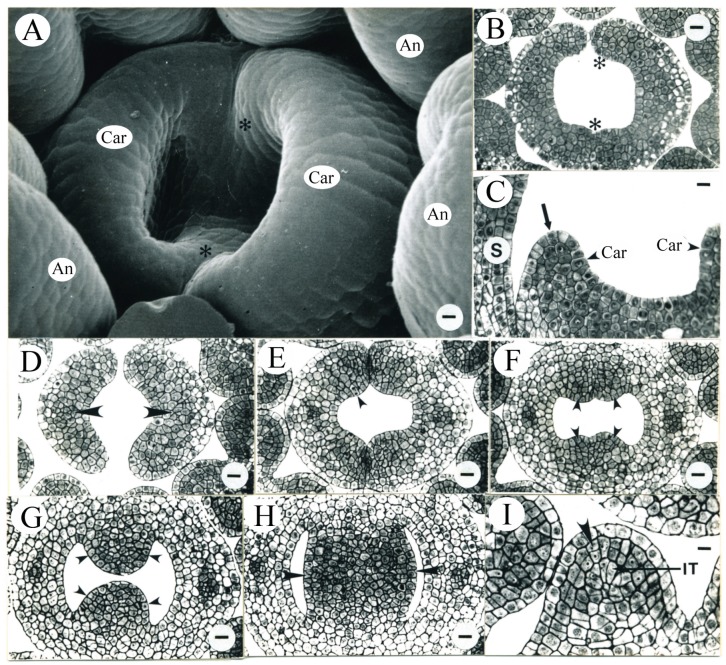
Tobacco pistil and anther development during the third day after ECP. (**A**) Representative SEM micrograph of a flower from the age interval 2.3 ± 1.2 days after ECP, showing two carpel primordia (Car) longitudinally elongating and arching inward. Asterisks identify the approximate location of the transverse section shown in (**B**). An, anther. Bar = 7.1 µm. (**B**) Transverse section of the carpel primordia seen in (**A**). Asterisks identify the approximate location of the section seen in (**A**). Bar = 19.4 µm. (**C**) Longitudinal section of the carpel primordia similar to those seen in (**A**). A recent periclinal division in L1 (arrow) is evident at the tip of one of the two carpel primordia (Car). The other carpel primordium is partially shown. S, stamen. Bar = 19.4 µm. (**D**–**H**) Serial transverse sections from a single pistil similar to that seen in (**A**). (**D**) Evident procambium in the top portion of carpels (arrowheads). Bar = 12.5 µm. (**E**) A recent cell division observed in L1 (arrowhead) in the margin of a carpel where two carpels have not fused. Bar = 23.1 µm. (**F**) Densely stained cells in the inner carpel wall below the fused margins of the carpels (arrowheads). Bar = 23.1 µm. (**G**) Densely stained cells in the central floral tissue enclosed inside the carpels (arrowheads). Bar = 20.5 µm. (**H**) The primordial placentae (arrowheads) initiating inside the carpels. Bar = 20.5 µm. (**I**) Transverse anther section showing a representative adaxial microsporangium from the age interval 3.0 ± 0.2 days after ECP. The majority of cells in L2 have completed a recent periclinal division (arrowhead) and the inner tapetum (IT) is now evident. Bar = 11.5 µm.

Greater histological differentiation was observed during the third day after ECP. The average pistil length reached 0.60 ± 0.06 mm, with the average flower age of 2.3 ± 1.2 days (N = 5). Two crest-like carpel primordia were arching inward and elongating around the circumference of the floral meristem (Figure 2A). The carpel primordia were horseshoe-shaped in transection, where they did not fuse laterally (Figure 2B). Recent periclinal divisions in the protodermal layer (L1) were observed at the tips of carpel primordia (Figure 2C). One major vascular strand evidently differentiated in each carpel (Figure 2D–H). The central floral tissue enclosed inside the carpels differentiated into placentae and was densely stained (Figure 2F–H), indicating that these cells were less differentiated and remained meristematic. The primordial placental tissues formed inside the carpels (Figure 2H). The average pistil length was increased to 0.63 ± 0.05 mm when the flower age was 3.0 ± 0.2 days (N = 3) after ECP.

**Figure 3:**
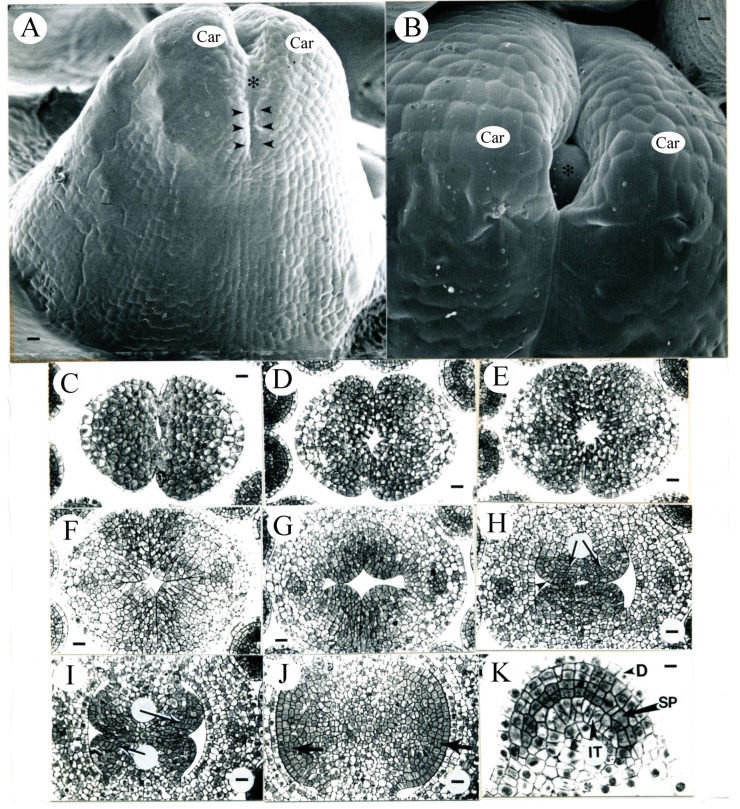
Tobacco pistil and anther development during the 5th day after ECP. (**A**) SEM micrograph of a representative tobacco pistil (lateral view) from the age interval 4.8 ± 1.3 days after ECP. The top portions of the carpels (Car) have not completely fused together (between arrowheads). The smooth surface (asterisk) visible between the carpel tips is an artifact of the epoxy cast. Bar = 16.3 µm. (**B**) SEM micrograph of a representative tobacco pistil (top view) from the age interval 4.8 ± 1.3 days after ECP. This tobacco pistil is not the same one shown in (13). The smooth surface (asterisk) visible inside the pistil is an artifact of the epoxy cast. Car, carpel. Bar = 6.4 µm. (**C**–**F**) Serial transverse sections of a pistil similar to those seen in (**A**) and (**B**), showing that the two carpels have not fused together in the top. Bars are 17.3 and 23.1 µm in (**C**) and (**D**–**F**), respectively. (**G**–**J**) Serial transverse sections of the pistil similar to those shown in (**A**) and (**B**), made in the region where the two carpels are fused together. Bar = 23.1 µm in (**G**). (**H**) Periclinal divisions observed (arrowhead) in the L1 layer of the central floral tissues enclosed inside the carpels with files of cells observed (arrows) below the L1 layer. Bar = 22.5 µm. (**I**) Files of cells (arrows) observed in the subepidermal layers within the placental tissue. Bar = 22.5 µm. (**J**) Recent periclinal divisions evident in L2 in the placenta (arrows). Bar = 22.5 µm. (**K**) Transverse section of a representative abaxial microsporangium from the age interval 4.8 ± 1.3 days after ECP. The sporogenous cells (SP) begin to appear cytologically distinct and are densely stained. IT, tapetum; D, protoderm. Bar = 10.7 µm.

During the fifth day (4.8 ± 1.3 days) after ECP, the average pistil length reached 0.92 ± 0.20 mm (N = 5). The top portions of the two carpels were in close contact with each other (Figure 3A–F). Cells in the outer or abaxial regions of the pistil were vacuolate, while those in the inner or adaxial regions of the pistil were generally densely stained (Figure 3C–G). Periclinal divisions were observed in L1 and deeper layers of the central floral tissues enclosed inside the carpels, creating files of cells (Figure 3H). Files of cells were also observed in the subepidermal layers of the placental tissue (Figure 3I). The placenta enlarged and recent periclinal divisions in L2 were evident (Figure 3J).

**Figure 4:**
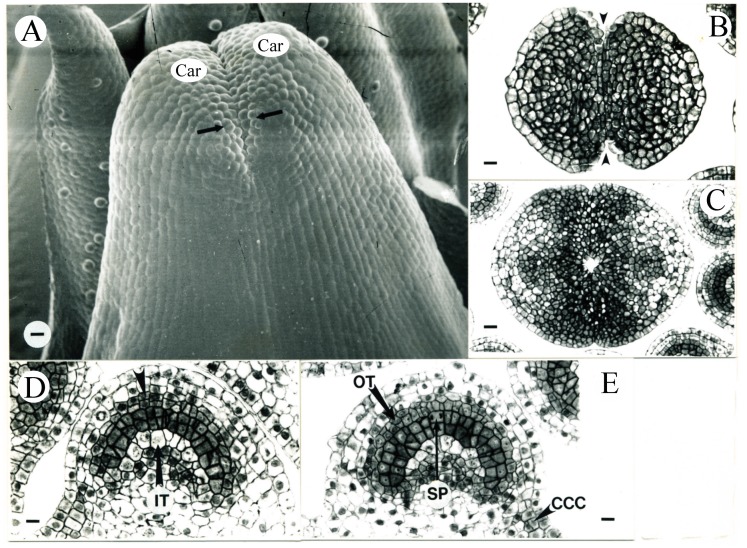
Tobacco pistil and anther development during the 6th day after ECP (**A**–**D**) and anther development during the 7th day (**E**) after ECP. (**A**) Carpels (Car) morphologically fusing in the top 5.4 days after ECP. The presence of the stigmatic papillar cells (arrows) indicates the differentiation of the stigma on the top portions of the carpels. Bar = 18 µm. (**B**,**C**) Transverse sections of the same pistil seen in (**A**), showing the carpel epidermal cells have not de-differentiated and carpel tips have not anatomically fused (between the arrowheads). Bars are 23.1 and 28.6 µm in (**B**) and (**C**), respectively. (**D**) Transverse section of an abaxial microsporangium from an anther in the same flower shown in (**A**). The outer tapetum (arrowhead) starts to differentiate. IT, inner tapetum. Bar = 12 µm. (**E**) Transverse section of a representative adaxial microsporangium from the age interval 6.1 ± 0.6 days after ECP. The outer tapetum (OT) is now evident. Sporogenous tissue (SP; arrow) is in mitosis. The cells in the circular cell cluster (CCC, arrowhead) are evident. Bar = 16.7 µm.

The carpel tips were undergoing postgenital fusion, also called ontogenetic or surface fusion, involving the union of tissues that arose as separate and discreate primordia, while the epidermal surfaces made contact and adhered; “fusion” was used to describe the adnation of cell walls rather than cytoplastic union [37] (Figure 4A–C). This postgenital fusion occurred about 5.4 days after ECP when the pistil was 1.31 mm (N = 1) in length, while the differentiation of the stigma in the top portion of carpels began. Minor vascular strands initiated within the carpel wall (data not shown). Two flowers were observed during the 7th day (6.1 ± 0.6 days) after ECP with pistil lengths of 1.40 and 1.67 mm, respectively.

**Figure 5:**
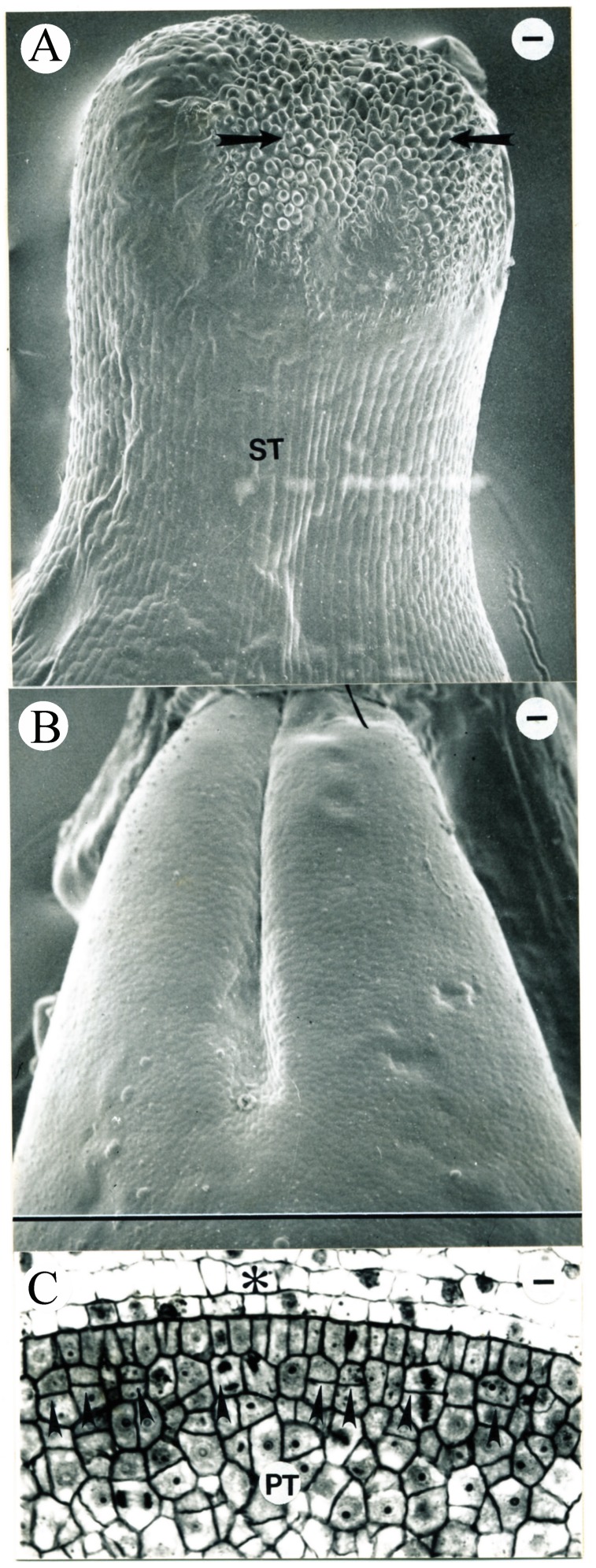
SEM micrographs and a transverse section of tobacco pistil during the 8th day after ECP. (**A**) During the age interval 7.6 ± 1.1 days after ECP, the papillar cells (arrows) are evident on the top of the stigma and the style (ST) begins to elongate. Bar = 20.7 µm. (**B**) SEM micrograph of the placenta in the same pistil seen in (**A**) with the carpel wall removed showing no morphological evidence of ovule initiation. The line indicates the approximate orientation of the corresponding histological section shown in (**C**). Bar = 18 µm. (**C**) Transverse section of a placenta at a developmental stage similar to that seen in (**B**). Periclinal divisions in L2 in the placenta are now common (arrowheads). The carpel wall is identified by an asterisk. Bar = 7.2 µm.

In flowers aged 7.6 ± 1.1 days after ECP, the pistils were 2.01 ± 0.51 mm (N = 8) in length. The formation of stigmatic papillae was evident (Figure 5A). The placenta greatly enlarged inside the carpels, while the ovule primordia were not morphologically evident (Figure 5B). Periclinal divisions in subepidermal cells were commonly observed in the placentae (Figure 5C), which contained 3–4 peripheral layers of cells densely stained, while cells below this zone became vacuolate. Branched vascular traces differentiated within the placental tissue (data not shown). The pistils were 2.60 ± 0.69 mm (N = 10) in length in flowers aged 8.9 ± 1.1 days after ECP.

**Figure 6:**
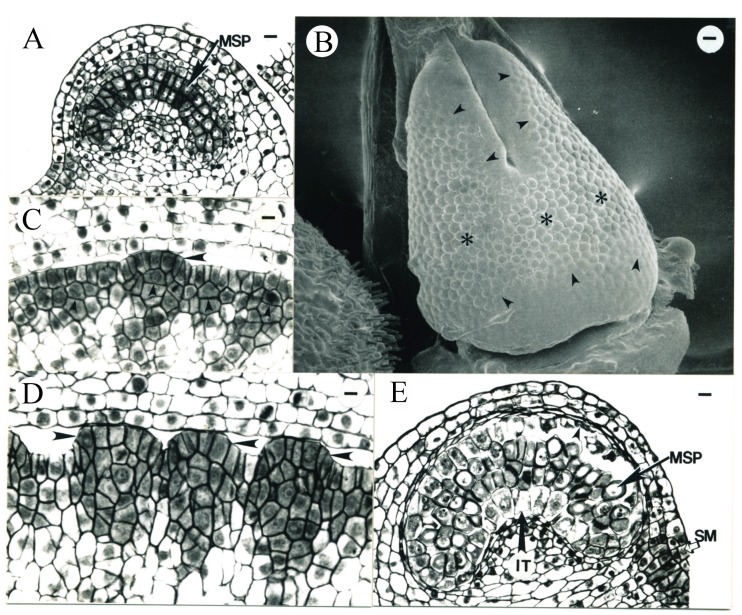
Tobacco anther development during the 9th day after ECP (**A**) and tobacco pistil and anther development during the 10th day after ECP (**B**–**E**). (**A**) Transverse section of a representative abaxial microsporangium from the age interval 8.9 ± 1.1 days after ECP. Cells in the sporogenous tissue are in prophase I of meiosis. MSP, microsporocyte. Bar = 17.3 µm. (**B**) SEM micrograph of ovules initiating in the placenta from the age interval 10.0 ± 0.5 days after ECP. Older ovule primordia are located in the middle portion of placenta (asterisks), while younger ones in the distal and the proximal regions on the placenta (arrowheads). Bar = 41 µm. (**C**) One ovule primordium (larger arrowhead) similar to those seen in (**B**). Ovule initiation is closely associated with the periclinal division in L2 cells in placenta (smaller arrowheads). Bar = 8.2 µm. (**D**) Three ovule primordia (arrowheads) similar to those seen in the middle portion of the placenta in (**B**) (asterisks). Bar = 9.7 µm. (**E**) Transverse section of a representative abaxial microsporangium showing the microsporocytes (MSP) when the average flower age is 10.0 ± 0.5 days after ECP. Some outer tapetal cells begin to degrade (arrowhead). Periclinal divisions in the CCC cells below stomium are evident. IT, inner tapetum; SM, stomium. Bar = 18.2 µm.

When the average flower age was 10.0 ± 0.5 days after ECP and the pistil length was 3.25 ± 0.30 mm (N = 4) in length, the ovule primordia initiated in the middle portion of the placenta and became evident at the distal and proximal edges (Figure 6B). Ovule initiation was closely associated with periclinal divisions in L2 (Figure 6C), while divisions in L1 remained anticlinal. In the placenta, 3–5 layers of cells below the ovule primordia were densely stained (Figure 6D). Ovule primordia protruded 1–2 layers of cells above the adjacent placental surface.

**Figure 7:**
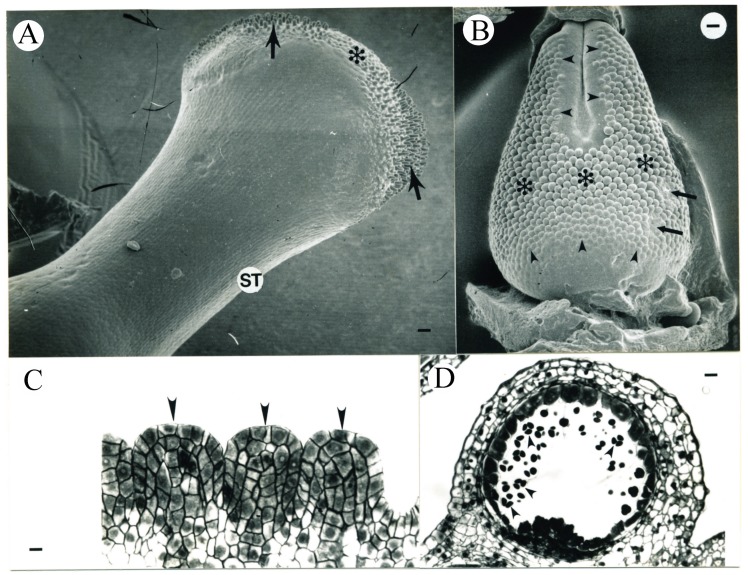
Tobacco pistil and anther development within 10.9 ± 1.1 days after ECP. (**A**) SEM micrograph of the stigma (asterisk) and the elongating style (ST). The papillar cells in the stigma differentiate on two distal areas (arrows) of the two carpels. Bar = 34.1 µm. (**B**) The placenta of the same pistil seen in (**A**) showing the enlarging ovule primordia. Larger ovules are in the middle portion of the placenta (asterisks), while smaller ovules and initiating ovule primordia are in the peripheral regions (arrowheads) of the placenta. Damage to the ovules (arrows) occurred when the pistil was removed from the mould. Bar = 65.2 µm. (**C**) Median longitudinal sections of three ovule primordia (arrowheads) from the placenta seen in (**B**). Bar = 8.2 µm. (**D**) Transverse section of an adaxial microsporangium showing the tetrads of microspores (arrowheads). Bar = 24 µm.

The pistil length reached 4.2 ± 0.3 mm (N = 3) when the average flower age was 10.9 ± 1.1 days after ECP. The style and stigmatic papillae further elongated (Figure 7A). Ovule initiation was observed across most of the placental surface (Figure 7B). The gradient of ovule initiation (comparison between Figure 6B and Figure 7B) resulted in the oldest ovule primordia in the center of the placenta, while the younger ones in the peripheral regions. Due to this gradient, the subsequent observations on ovule development were consistently made from the oldest ovules located in the middle portion of the placenta, unless otherwise noted. The oldest ovule primordia appeared as finger-liked protrusions and were 5–7 layers of cells above the adjacent epidermis of the placenta (Figure 7C). All of the cells in the ovule primordia were densely stained, while the megasporocytes were not evident.

**Figure 8:**
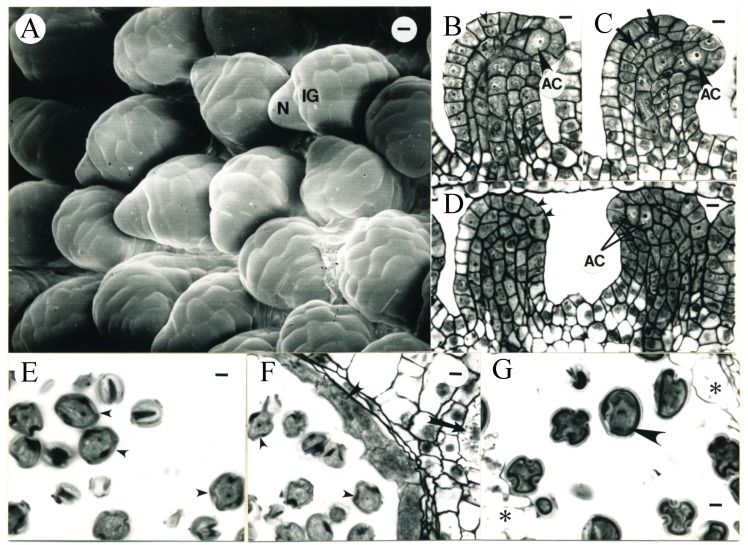
Tobacco ovule and anther development during the 13th day after ECP (**A**–**F**) and anther development during the 14th day after ECP (**G**). (**A**) SEM micrograph of representative ovules from the age interval 12.9 ± 1.2 days after ECP. The distal end of each ovule begins to recurve. Nucellus (N) and integument (IG) are evident. Bar = 6.7 µm. (**B**–**D**) Longitudinal sections of ovules similar to those seen in (**A**). (**B**) The evident archesporial cell (AC, larger arrowhead) in the subepidermal layer of the ovule. Integument initiation is associated with periclinal divisions in L1 of the ovule (smaller arrowhead). Bar = 7.6 µm. (**C**) Recent periclinal divisions in L2 at the recurving end in the ovule (arrows). AC, archesporial cell. Bar = 7.6 µm. (**D**) Two ovules showing twin archesporial cells (AC) observed in a single ovule and periclinal divisions (arrowheads) observed in the L1 layer of the other ovule. Bar = 8.2 µm. (**E**) Microspores (arrowheads) shown in the anthers with the average flower age of 12.9 ± 1.2 days after ECP. Bar = 5.5 µm. (**F**) The degrading outer tapetum (larger arrowhead) with microspores (smaller arrowheads) observed as those in (**E**). CCC cells contain crystals (arrow) and have lost staining color. Bar = 6.5 µm. (**G**) Mitotic divisions in anaphase (arrow) observed in microspores with the average flower age of 14.0 ± 1.2 days after ECP. Remnants of tapetum (asterisks) are still present. Bar = 6.1 µm.

In about 13 days (12.9 ± 1.2 days) after ECP, the pistil length reached 6.6 ± 1.2 mm (N = 8), the ovules enlarged, and the distal ends of the oldest ovules began to recurve (Figure 8A). The differentiation of integument in some ovules was evident (Figure 8A), revealed anatomically by periclinal divisions in both L1 and L2 (Figure 8B–D), while about 7–8 files of cells in each ovule were observed in the median longitudinal sections. Some epidermal cells in the ovule became vacuolate. Fewer periclinal divisions were observed in the inner side of curving ovule primordia in the median longitudinal sections. Subepidermal archesporial cells became distinct from the surrounding nucellar cells due to their bigger size and light staining color (Figure 8B–D). Most ovule primordia contained only one archesporial cell which would function directly as a megasporocyte. Two archesporial cells were observed in 20 out of 450 ovules examined (<5%; Figure 8D). Most of the placental cells were vacuolate except for those in the vascular bundles (data not shown). The pistil reached 10.4 ± 1.1 mm (N = 4) in length in about two weeks (14.0 ± 1.2 days) after ECP (Figure 8G).

**Figure 9:**
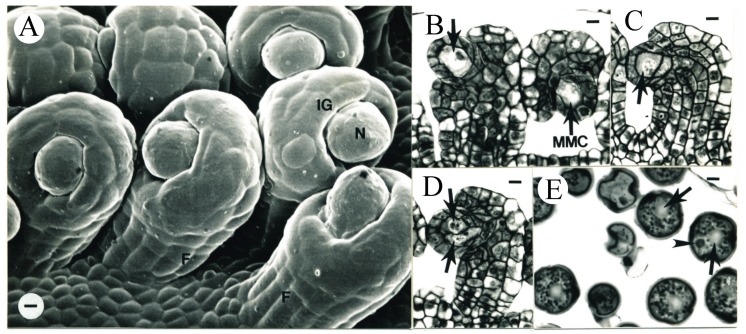
Tobacco ovule development during the 15th day after ECP (**A**–**D**) and anther development during the 16th day after ECP (**E**). (**A**) SEM micrograph of representative ovules from the age interval 14.4 ± 1.3 days after ECP. The integument (IG) surrounds the nucellus. The funiculus (F) has differentiated. Bar = 6.7 µm. (**B**–**D**) Longitudinal sections of ovules similar to those shown in (**A**). (**B**) Megasporocytes in meiosis (arrowhead). Bar = 10.3 µm. (**C**) Two megasporocytes observed in one ovule. The smaller one is behind the larger one (arrow). Bar = 10.3 µm. (**D**) Two megasporocytes observed in one ovule (arrows). Bar = 10.3 µm. (**E**) Evident pollen grains each containing a vegetative cell (arrowhead) and a generative cell (arrows) with the average flower age of 16.0 ± 0.4 days after ECP. Bar = 6.1 µm.

Shortly after two weeks (14.4 ± 1.3 days) after ECP, the pistil length reached 10.9 ± 1.5 mm (N = 5). One single integument almost enclosed the nucellus (Figure 9A). Vascular tissues differentiated in the funiculi of ovules. Morphologically, the developmental gradient across the placenta established at the time of ovule initiation (Figure 6B and Figure 7B) maintained. The ovules in the middle portion of placenta curved down, the integuments almost surrounded the nucelli, and the megasporocytes in the subepidermal layer were in prophase I (Figure 9B–D). Two megasporocytes within a single ovule were observed at a low frequency (<5%; Figure 9C,D) at this stage. In the peripheral regions of the placenta, ovules began to curve down and the integuments initiated. Although the megasporocytes in some of these ovules were already in prophase I, the archesporial cells in some other ovules were similar to those seen in ovules in younger flowers (e.g., Figure 8B–D), suggesting that the ovules in the peripheral regions of the placenta were about 1.5 days younger than those in the middle portion of the placenta and the megasporogenesis did not occur simultaneously in the ovary due to the different rates of ovule development. When flowers were 16.0 ± 0.4 days after ECP, the pistils were 17.0 ± 3.8 mm (N = 3) long (Figure 9E).

**Figure 10:**
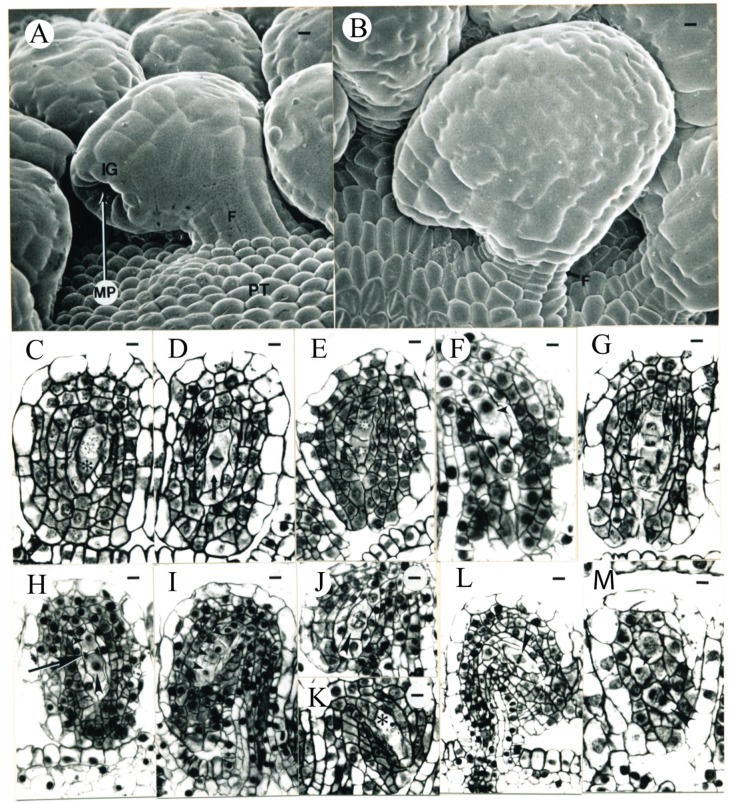
Tobacco ovule development 16.2 ± 0.4 days after ECP. (**A**) SEM micrograph of the anatropous ovules from the peripheral region of the placenta. F, funiculus; IG, integument; MP, micropyle; PT, placental epidermis. Bar = 7.0 µm. (**B**) SEM micrograph of the anatropous ovules from the middle portion of the placenta. F, funiculus. Bar = 7.0 µm. (**C**–**I**) Transverse sections of ovules similar to the one seen in the middle of (**B**). (**C**) Megasporocyte (asterisk) in prophase I. Bar = 8.1 µm. (**D**) Megasporocyte (arrow) in metaphase I. Bar = 8.1 µm. (**E**) Two dyad cells in prophase of meiosis II (arrowheads). Bar = 10 µm. (**F**) Two dyad cells asynchronous in meiosis II. The micropylar dyad cell is indicated by a larger arrowhead, while the chalazal dyad cell is indicated by a smaller arrowhead. Bar = 7 µm. (**G**) Two dyad cells asynchronous in meiosis II. The micropylar dyad cell is in metaphase II (larger arrowhead), while the chalazal dyad cell is indicated by a smaller arrowhead. Bar = 8.1 µm. (**H**) Micropylar dyad cell in metaphase II (larger arrowhead). One of the two chalazal megaspores has degraded (arrow) and is flanked by the micropylar dyad cell and the other chalazal megaspore (smaller arrowhead). Bar = 10 µm. (**I**) Linear tetrad of 4 megaspores (arrowheads) in the ovule. Bar = 10 µm. (**J**–**M**) Transverse sections of ovules similar to the one seen in the middle of (**A**). (**J**,**K**) Megasporocytes in prophase I (arrowhead, asterisk). Bar = 9 µm. (**L**) Megasporocyte in metaphase I (arrowhead). Bar = 11.5 µm. (**M**) Two dyad cells (arrowheads). The micropylar dyad cell is identified by the larger arrowhead. Bar = 7.5 µm.

The ovules of anatropous type almost fully formed during the 17th day (16.2 ± 0.4 days) after ECP. Two flowers were observed with pistil length of 17.5 and 20.5 mm, respectively. Most of the epidermal cells in the ovules changed from a rectangular to an irregular shape that was evident in the mature seed coats (Figure 10A,B). The developmental gradient, following the pattern of ovule initiation (Figure 6B and Figure 7B), was still present and consequently, the ovules were temporally, morphologically, and histologically different from each other within a single placenta depending on where the ovules were located. At this age, most of the epidermal cells in ovules were vacuolate, while the megasporocytes were found in either metaphase I (Figure 10C,D), dyads (Figure 10E), metaphase II (Figure 10F–H), or tetrads (Figure 10I). Meiosis I was almost an equal division (Figure 10E,F), generating two dyad cells of almost the same size. Meiosis II within a single ovule could be either synchronous (Figure 10E) or asynchronous (Figure 10F–H). In the asynchronous development, the micropylar dyad cell would divide either before (Figure 10F) or after (Figure 10G,H) the chalazal dyad cell. Linear tetrads were the only arrangement of megaspores observed in this study (Figure 10I). The shape of some ovules in the peripheral regions of the placenta were not the normal anatropous type (Figure 10A), perhaps due to both the temporal delay in ovule initiation and the physical pressure of the carpel walls. These ovules (Figure 10J–M) were less mature than those in the middle portion of the placenta (Figure 10C–I). Some of the immature ovules still contained a single megasporocyte in prophase I (Figure 10J–L). Dyads in telophase II were observed (Figure 10M), but no tetrads were observed in these ovules, suggesting that the youngest ovules in the peripheral regions of the placenta were about 1.8 days younger (e.g., Figure 9B,C) than the ovules in the middle portion of the placenta. The second megasporocyte (Figure 9C,D), occasionally observed within a single ovule, was in meiosis.

**Figure 11:**
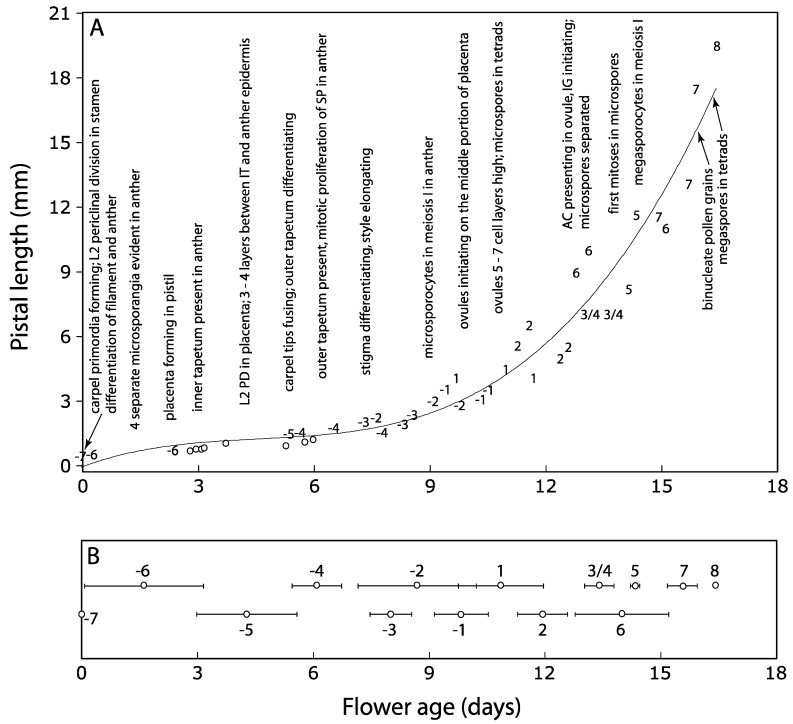
Tobacco pistil and stamen development over time. Plotted values (–7 through 8) are the developmental stages, as previously described by Koltunow et al. (1990). Flower ages are estimated by the PI approach. (**A**) Flowers are staged by the major developmental events and morphological characteristics in anther and pistil [30]. The correlational relationships between the pistil length and time was calculated as Y = 0.0091X^3^ − 0.126X^2^ + 0.6697X − 0.1001, where Y was the pistil length and X was the time (R^2^ = 0.9449). PD, periclinal division; SP, sporogenous cell; AC, archesporial cell; IG, integument; 3/4, Stages 3 or 4. Eight flowers indicated by the open circles are categorized as Stage –5 based on the characteristics “inner tapetum present, and no outer tapetum present” in the anthers. However, these flowers are also categorized into Stage –6 based on the morphological characteristics of “carpels not fused” in pistil. (**B**) Summary of the average ages (with standard deviations presented horizontally) of tobacco floral developmental stages. The eight flowers presented by the open circles in (**A**) are included in Stage –5 in the calculation of average ages.

Major morphological and anatomical observations during the female and male organ development in tobacco are summarized in Figure 11. It took about 17 days for the flowers to develop from Stage –7 (ECP) to Stage 8 (megaspores in tetrads and bicellular pollen grains in anthers) based on [30] (Figure 11A). These results showed that the duration of individual floral developmental stages was not uniform (Figure 11B). Although the sexual maturation was synchronous at the level of the whole flower, evident temporal distinction existed between megasporogenesis and microsporogenesis. It was sometimes problematic to assign stages to young floral primordia, especially during Stages –6 and –5 based on [30] because of the lack of correspondence between diagnostic traits observed in pistil and stamen. For example, both sets of characteristics defining Stage –6 (unfused carpel primordia) and Stage –5 (anther tapetal cells present) were observed simultaneously in the same flowers in our study (Figure 2I and Figure 3A,B,K).

## 3. Discussion

We here report the temporal patterns of the female floral organ (pistil) development in comparison to the anther development within single flowers using corolla growth analysis in tobacco. A total of 42 flower primordia or flowers was aged spanning a 17-day interval, starting with flower primordia containing the early carpel primordia (ECP) and the anther primordia and filaments. At the end of this interval, megasporocytes reached the tetrad stage of meiosis and pollen grains reached the bicellular stage.

### 3.1. Photoperiod Cycle Affects the Floral Initiation and Development

One aspect of developmental biology is to use the quantitative analysis of growth and development to determine the timing of developmentally important events. PI is used as a convenient measure to construct the temporal sequences of ontogenesis and to reveal the temporal patterns of ontogenesis among floral organs. PI determines the developmental age of a plant organ in terms of plastochron, which is the period of time between successive plant organs reaching a conveniently measurable reference length. When the plastochron is of constant duration, its rhythm can be utilized as a unit of time.

We established a PI based on a chosen reference length in pistil to calculate the average plastochron in six plants, as reported previously [38]. The average cyme plastochrons obtained in this study increased gradually from positions 2 through 8. This pattern is substantially comparable with that reported by [38] for the same tobacco cultivar grown under a similar light–dark cycle (8-h light and 16-h dark) and temperature (25 °C). However, these results are different from another pattern previously observed also for the same tobacco cultivar [6], where the average cyme plastochrons fluctuated and ranged between 1.04 and 2.48 days and were generally shorter than those of the corresponding flower positions in our study. This discrepancy is most likely due to the change of light–dark cycle from continuous illumination [6] to the photoperiod cycle of 9-h light and 15-h dark in our study. Although the rate of flower initiation in the cyme does not have to be correlated directly with the growth rate of individual flowers, the duration of specific floral developmental stages changed almost proportionally with the change in the light cycle in the present study. For example, the average flower age when meiosis began in the anther was 8.9 ± 1.1 days after ECP. The same window of anther development was 3.6 days (from 3.0 to 6.6 days) under continuous illumination [6], which was a decrease of about 60%, corresponding to a decrease of 62.5% in the light cycle in our study with a photoperiod of 9-h light and 15-h dark. Furthermore, studies showed different effects of temperature on time of floral initiation under different photoperiods. For example, it was reported in *Sorghum bicolor* that floral initiation was delayed under a 17-h photoperiod, as compared to a 10-hour photoperiod [39], indicating the potential interactions between temperature and photoperiod in affecting the floral initiation.

### 3.2. Unequal Duration of Male and Female Floral Organ Developmental Stages

Koltunow et al. [30] used floral bud lengths, major developmental events, and morphological markers in the pistil and stamen to divide floral development through anthesis into 19 stages in *Nicotiana tabacum*. Our results showed that for many stages, the duration of each stage is not equal. For example, in some flowers, carpel tips were not fused (characteristics for Stage –6 in [30]), but inner tapetum was formed in the anthers (characteristics for Stage –5). These results indicated that the process of carpel fusion is too protracted in tobacco to be a good morphological marker for staging primordia during early floral development. Therefore, we speculate that the staging schemes along a linear scale do not imply an equally linear time course for ontogeny during floral development in tobacco. A strong correlation between flower bud length and anther development in lily was revealed by studying the rate of floral development using flower bud length and an average flower bud growth rate [32], while a correlation among external flower development and the time and pattern of reproductive organogenesis was established in *Capsicum annuum* [40]. Furthermore, it was reported that the developmental variations that affect the phenotypes of the female and male floral organs may be involved in the modifications of gene functions [10]. In our study, we provided the major morphological and anatomical characteristics during the early stages of pistil and stamen development over the same time scale. These characterizations establish a solid foundation for future studies to identify the correlated developmental stages in these two floral organs.

### 3.3. Carpel Development

Hicks [41] demonstrated that carpel initiation was associated with both the periclinal divisions in L2 and the more randomly oriented divisions in the corpus zone (L3) in tobacco, while the protoderm of each carpel primordium was basically expanded by anticlinal divisions in L1. The same type of carpel initiation and growth pattern were also reported in many species of plants, including *Arabidopsis* [42,43]. Furthermore, studies showed that in both tobacco [41] and *Datura* [44], the carpel initiation and early development depended primarily on the cell division activity of L3, while cells from L1 and L2 only divided anticlinally. Genetic studies have shown that in chimeras of *Arabidopsis* that differed in the number of carpels, the carpel number during carpel initiation was determined by the genotype of cells occupying the L3 of the meristem [45] because cells in different layers of the meristem communicate with each other during floral organ initiation. Our results showed that the periclinal divisions in the L1 layer at the tips of carpel primordia were observed, even though these divisions were rare. The similar results were also reported in a previous study showing a low ratio of periclinal to anticlinal divisions in L1 of the tobacco ovary [46]. Therefore, our results indicate that both anticlinal and periclinal divisions in L1 contribute to the carpel early development in tobacco.

### 3.4. Placenta Development and Ovule Differentiation

Generally, the periclinal divisions in L2 in the placenta marked the beginning of ovule initiation. Placenta development characterized by periclinal divisions in L2 was also reported in *Lythrum salicaria* [47]. However, in *Datura* [44], the placenta was built primarily by cells from L3, and the contribution of L1 and L2 was limited to one layer each. In tobacco, we speculate that the long duration of periclinal divisions in L2 in the placenta probably contributes as much to placenta growth as it does to just the indication of the beginning of ovule initiation. Furthermore, the interval from 7.6 ± 1.1 days to 10.0 ± 0.5 days after ECP might be a crucial phase for ovule initiation. Future studies at genetic and molecular levels may focus on these developmental stages to reveal the molecular mechanisms controlling the ovule initiation and development. In tobacco, it was suggested that there was a developmental window, beginning at the initiation of ovule primordia, when the placenta did not become committed to ovule differentiation [48]. We observed that ovule initiation was closely associated with periclinal divisions in L2 in the placenta and a group of densely stained cells below each ovule primordium, suggesting that important molecular factors controlling the ovule initiation may be expressed during this interval (about 2.4 days). The commitment to ovule growth may occur just prior to the development of the nucellar mass and the appearance of the integument, while the factors involved in the commitment to ovule growth have not been identified [49,50].

### 3.5. Formation of Megasporocytes

Generally, only one archesporial cell differentiated in each ovule, while the differentiation and development of two or more adjacent archespores are not exceptional. Two or more archesporial cells or megasporocytes have also been reported in many species of plants [51,52,53,54,55,56]. Surprisingly, it was reported that each ovule had only one archesporial cell in *Nicotiana tabacum* [53], which enlarged and functioned directly as a megasporocyte. Interestingly, Goodspeed [53] observed twin embryo sacs in a single ovule in tobacco. The twin embryo sacs were probably formed from twin megasporocytes that were probably overlooked by Goodspeed [53] because the formation of two such cells was rare, as confirmed by our results, which showed that two archesporial cells or megasporocytes were observed within a single ovule at a low frequency, while only one went through meiosis. Nonomura [57] suggested that multiple cells in L2 had the potential to become a megasporocyte, while multiple megasporocyte-like cells were reported in mutants of the THO/TREX complex genes in *Arabidopsis* [58]. It was suggested that the position of the putative megasporocyte may be an important factor in cell specification in *Arabidopsis* [59]. Furthermore, the molecular factors determining the identity of megasporocyte and the molecular mechanisms controlling the selection of ovular cells as the archesporial cells have been explored in various species [60], while both auxin and cytokinin have been demonstrated to play crucial roles in the ovule primordia development [61].

### 3.6. Pistil and Stamen Development in Tobacco

By comparing some of the organelles and macromolecules between megasporogenesis and microsporogenesis, Willemse and van Went [62] reported that the subcellular and functional activities during megasporogenesis and microsporogenesis were comparable until the megaspores and microspores reached their functional stages in plants. However, the developmental patterns of megasporogenesis and microsporogenesis are not generally synchronous. For example, meiosis in anthers was about 19 days earlier than that in ovules in lily [31]. Morphological evidence indicated that the interval from stamen initiation to carpel initiation was about 2.3 days (under continuous illumination) [6]. If we increase this interval by 62.5% for the decreased photoperiod used in the present study, then the interval from stamen initiation to carpel initiation is estimated to be about 5.7 days. Our results showed that meiosis in anthers was estimated to be about 5.5 days earlier than that in ovules. When the ovules reached the tetrad stage, the anthers already reached the bicellular pollen stage (Stage 8 in the work of [30]). It was also reported in *Paspalum rufum* that both the female and male reproductive development were equally synchronized in diploids, while the megasporogenesis was delayed with respect to microsporogenesis in tetraploids [63]. Nevertheless, these temporal distinctions between megasporogenesis and microsporogenesis do not subsequently affect the normal self-pollination and self-fertilization in this self-compatible cultivar of tobacco, because the sexual maturation is synchronous at the level of the whole flower. The staggered development of megasporogenesis relative to microsporogenesis is eliminated at anthesis when pollen tube growth proceeds through the transmitting tissue in the style, while the megagametophytes are forming in the ovules.

## 4. Materials and Methods 

### 4.1. Growth of Tobacco Plants

**Figure 12:**
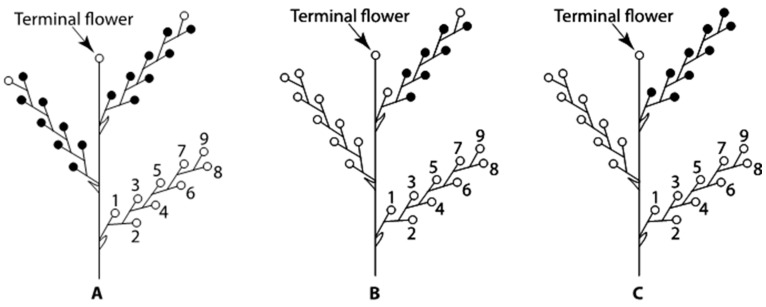
Scheme of *Nicotiana tabacum* cymes indicating the flowers and cymes collected for corolla growth analysis (**A**), scanning electron microscopy and histological analyses (**B**), and supplementary materials for histological analysis (**C**). Flower positions 1 through 9 are marked on one of the three cymes. (**A**) Corolla growth was measured on the first eight flowers (closed circles) to open on two cymes from each of the six plants in Group 1 tobacco plants. (**B**) Scanning electron microscopy and histological analyses were conducted on the seven flowers or flower buds (closed circles at positions 2 to 8) on one cyme from each of the six plants in Group 2 tobacco plants. (**C**) One cyme from each of the six plants in Group 3 tobacco plants was collected as supplementary materials for histological analysis. The corolla lengths of the first flowers in these six cymes were the same as those of the six cymes in Group 2 plants.

Cultivation of tobacco plants (*Nicotiana tabacum* L. cv. Xanthi) was similar to that previously described [6] with a photoperiod cycle of 9-h light and 15-h dark being used in this study instead of continuous illumination. The length change of the light-dark cycle enabled us to compare its effects on the plastochrons and the temporal characterization of floral organ development under different growth conditions. The temperature (23 °C) and light-dark cycle were maintained for the entire experiment, starting from the seed germination in a growth chamber. A total of 18 seedlings with 3–5 visible leaves, divided into three equal groups (Groups 1, 2, and 3) for different experiments (Figure 12), were transplanted to 9-cm square plastic pots and transferred to a walk-in growth chamber. When the leaves of each seedling were about 5 cm in length and almost covered the surface of the pot, the seedlings were then transplanted to 1-gallon plastic pots. Plants were watered with distilled water as needed to keep the leaves from wilting and fertilized once a week with Peter’s 20:10:20 Peat-Lite Special (21 g/L; W.R. Grace, Fogelsville, PA, USA).

### 4.2. Determination of Inflorescence Plastochron

Corolla growth of the six plants in Group 1 was studied as described by Hill and Malmberg [38]. Corolla length of the first eight flowers on a total of 12 cymes (two cymes on each of the six plants) was measured daily to open (Figure 12A). A corolla reference length of 3.0 cm was used in the calculation of cyme plastochrons (the interval between the initiation of successive flowers within a cyme), as previously reported [38]. Statistical analyses were performed with Statistical Analysis System (SAS; release 6.03 edition, SAS Institute Inc., Cary, NC, USA). Specifically, as shown by Hill and Lord [3]:

$$t_{Ri}=t_{2}-\left(t_{2}-t_{1}\right)\frac{\ln\left[L\left(n_{i},t_{2}\right)\right]-\ln\left(R\right)}{\ln\left[L\left(n_{i},t_{2}\right)\right]-\ln\left[L\left(n_{i},t_{1}\right)\right]}$$

where *i* = the node number, beginning with a basal node and increasing in the acropetal direction, *t_Ri_* = time when the organ (corolla) at node *i* equals the reference length (*R* = 3.0 cm), *t*_1_ = time of initial observation of the organ at node *i*, *t*_2_ = time of final observation of the organ at node *i*, *n_i_* = the organ at the *i*th node, which was the youngest organ whose length at *t*_2_ was larger than or equal to *R*, *L*(*n_i_*, *t*_1_) = length of the organ at node *i* at *t*_1_, and *L*(*n_i_*, *t*_2_) = length of the organ at node *i* at *t*_2_.

Flowers and flower buds from six cymes removed from Group 2 plants were fixed in 50% ethanol-acetic acid-formalin (90:5:5 by volume) (FAA) for histological studies (Figure 12B). The sampling of the cymes and flowers on the specific cymes and subsequent calculations of PI and estimation of flower bud ages on these six cymes on a common time scale were conducted as previously described [6,38]. The corolla lengths of the first flowers on each of these six cymes were 2.0, 2.5, 3.0, 3.5, 4.0, and 4.5 cm, respectively. A corolla reference length of 1.5 cm was used in the calculation of PIs for the fixed materials. A total of 42 flowers or flower primordia, from positions 2 to 8 in each cyme, were aged on the same time scale, with the youngest flower set as zero. Specifically, the PI was previously defined [33] and calculated as

$$PI=n+\frac{\ln(L_{n})-\ln\left(R\right)}{\ln(L_{n})-\ln(L_{n+1})}$$

where *n* = node number whose organ (corolla) was just longer than the chosen reference length (*R*) and the node number began with a basal node and increased in the acropetal direction, *R* = reference length (1.5 cm) chosen in the exponential phase of organ growth, *L_n_* = length of an organ just longer than the chosen reference length, and *L_n+_*_1_ = length of an organ at the next youngest node after node *n*.

A PI value of 3.0 of the cyme with the corolla length of the first flower on this cyme equal to 4.5 cm was set equal to zero on the time scale. Flowers on a particular cyme were then aged in the following manner: a cyme with a particular PI value (i.e., PI = 1.78) was 22% of plastochron 2 and 100% of plastochron 3 away from plastochron 3 (point zero on the time scale). Thus, the flower at position 2 was at time = 0 − [(22%)*(plastochron 2) + (100%)*(plastochron 3)] on the time scale. The younger flowers (at positions 3–8) were aged by subtracting the appropriate average plastochron from each successive position so that their ages were expressed as negative values. Similarly, the older flowers were aged by adding appropriate average plastochron to each successive position. This operation was repeated for all six cymes to reconstruct the earliest phase of flower growth over time. A plot of pistil length over time with major floral developmental events highlighted was constructed with the points of time shifted along the time scale to position the youngest flower of all six cymes (i.e., the most negative flower age) as zero. Gynoecium lengths larger than or equal to 3 mm were measured from the top of the pistil to the base of the floral receptacle using calipers. Pistil lengths shorter than 3 mm were obtained from the serial 3-µm sections using an ocular micrometer.

### 4.3. Microscopy and Histology

Prior to tissue fixation for histological studies using light microscopy on flowers of plants in Group 2, moulds of pistils or flower primordia were made using the dental impression plastic (Cutter Perfourm Light, Miles Inc., Germany) generally following the method described by Green and Linstead [64]. The epoxy casts subsequently replicated from these moulds were observed with the scanning electron microscope (SEM, Amray, 1000), making it possible that both the morphological and histological observations were made on the same tissue sample. Large pistils with two carpels fused at the top portions were first dissected longitudinally, and then the moulds were made of the placenta inside one carpel. The remaining carpel tissue was then fixed in FAA for histological sectioning. The entire pistil primordium in small carpel primordia was used to make the mould without being dissected prior to being fixed in FAA for histological sectioning.

To supplement the results from Group 2 plants, an additional six cymes from Group 3 plants were fixed directly in FAA for histological studies (Figure 12C). The corolla lengths of the first flowers in these six cymes were similar to those of the six cymes from Group 2 plants. The serial 3-µm sections of flowers from both Groups 2 and 3 were stained and observed, and representative views were photographed as previously described [6]. No histological differences were observed during floral development between plants in Groups 2 and 3. Pistil and stamen development in the aged flowers from positions 2 through 8 in these two groups was reported. The flower staging systems reported by Koltunow et al. [30] and Hill and Malmberg [6], combined with the plastochron aging method by Hill and Malmberg [6,38], were used to describe the rates of early floral development. The average age and pistil length of flower primordia at different developmental stages previously established for tobacco [30] were calculated.

## 5. Conclusions

We summarized the major anatomical, morphological, and embryological characteristics of the pistil and the anther development of *Nicotiana tabacum* cv. Xanthi over a real time scale. Flower ages spanned a 17-day interval, starting with flower primordia containing the ECP and anther primordia to the tetrad stage of meiosis in megasporocytes and the bicellular stage in pollen grains. Our results revealed various differences in the cellular basis of tobacco pistil development compared to previous reports in tobacco, *Datura*, and other species of plants. These differences in the developmental stages between female and male floral organs represent the potential phases evolutionary selection might act on, ultimately determining the evolution of mating systems. Future studies are necessary to reveal the developmental and molecular mechanisms controlling the rate of flower initiation and development under various environmental conditions.

## Figures and Tables

**Table 1:** The average cyme plastochron (in days) vs. flower position in tobacco. Data of corolla length used to calculate the cyme plastochrons were collected from the six plants in Group 1 (12 cymes) with a photoperiod cycle of 9-h light and 15-h dark. A corolla reference length of 3.0 cm was used in the calculation of these seven cyme plastochrons.

Flower Position	Plastochron ± Standard Deviation
2	1.45 ± 0.26
3	1.57 ± 0.31
4	1.84 ± 0.10
5	2.06 ± 0.35
6	1.98 ± 0.19
7	2.55 ± 0.38
8	2.85 ± 0.45

**Table 2:** Plastochron indices (PIs) of the six cymes in Group 2 plants. A corolla reference length of 1.5 cm was used to calculate these six plastochron indices. Values in parentheses are the corolla lengths of flowers at position 1 in each of these six cymes.

Cyme #	PI
1 (2.0 cm)	1.61
2 (2.5 cm)	1.78
3 (3.0 cm)	2.17
4 (3.5 cm)	2.54
5 (4.0 cm)	2.65
6 (4.5 cm)	3.00

**Table 3:** Temporal comparisons of the stamen and anther development in tobacco between the current and two previous studies [6,30].

Anther Age (Days after ECP) and Developmental Characterization	Stage Defined in [30]	Age (Day) Reported in [6]
0 (Figure 1A,B): Filaments and anthers differentiated in stamen primordia. Periclinal divisions in the hypodermal layer (L2) in the anther were observed. A single procambial strand was shown in the region of the anther connective.	Stage –7	3.0
1.3 ± 1.5 (Figure 1C): Four separate microsporangia were evident. Periclinal divisions of L2 cells were observed.	Stage –6	3.7
3.0 ± 0.2 (Figure 2I): The tetrasporangiate anthers was evident. Most of the archesporial cells completed the first round of periclinal divisions, while the inner tapetal cells were identified beneath a layer of archesporial cells. The majority of cells in L2 completed a recent periclinal division.	Stages –6, –5	4.4
4.8 ± 1.3 (Figure 3K): The anther generally contained 3–4 layers of cells between the inner tapetum and the epidermis. The region of 1–2 layers of sporogenous cells immediately adjacent to the inner tapetum became cytologically distinct, while no outer tapetum differentiated.	Stage –5	4.7–5.0
5.4 (Figure 4D): The anther contained 3–4 layers of cells between the layer of sporogenous tissue and the epidermis. The outer tapetum differentiated as 1–2 layers of cells immediately outside the sporogenous tissue.	Stages –4, –3	5.9
6.1 ± 0.6 (Figure 4E): Sporogenous cells in anthers were in mitotic proliferation. The outer tapetum was evident as a layer of 1–2 smaller cells immediately outside the sporogenous layer. Some cells in the stomium and circular cell cluster (CCC, a group of cells underneath the stomium layer that accumulate crystals and degenerate when the two microsporangia fuse into one locule prior to anther dehiscence) were densely stained. Recent periclinal divisions were observed in the CCC region without any crystal accumulation in these cells.	Stages –3, –2	6.0
8.9 ± 1.1 (Figure 6A): Microsporocytes entered prophase I of meiosis. More periclinal divisions were observed in the cells of CCC and some CCC cells contained crystals.	Stages –2, –1	6.6
10.0 ± 0.5 (Figure 6E): Microsporocyptes were separated from the anther wall. Periclinal divisions were observed in stomium cells, while some outer tapetal cells began to degrade.	Stage –1	N/A
10.9 ± 1.1 (Figure 7D): Microspores were in tetrads and tapetum was degenerating.	Stage 1	N/A
12.9 ± 1.2 (Figure 8E,F): Microspores were separated from each other. Cells of CCC contained crystals and lost staining. Tapetal cells were degenerating.	Stages 2–4	N/A
14.0 ± 1.2 (Figure 8G): Microspores were in mitotic divisions in anaphase. Tapetal cells were further degraded, while the remnants of the tapetum were still present. Secondary thickening on the cell walls of the endothecial layer in the anthers was observed, while the CCC cells began to degrade and some stomium cells collapsed.	Stages 5–6	N/A
16.0 ± 0.4 (Figure 9E): Both the vegetative and generative cells were observed in pollen grains. The cells of CCC completely degraded, while the two pollen sacs fused into one locule. Most connective cells were still intact, while the tapetal cells almost completely degraded. The anthers did not open yet.	Stages 7–8	
